# Book Reviews

**Published:** 1919-02

**Authors:** 


### BOOK REVIEW.

Manual of Vital Function Testing Methods and Their Interpretation: Second Revised and Enlarged Edition. Wilfred M. Barton, M. D. 12mo. of 318 pages. Boston: Richard G. Badger, Publisher, 1917.

Beginning with the liver and ending with ductless glands, the book quite thoroughly discusses the many tests which are of possible clinical value. The book is admirably arranged supplying, where knowledge remains incomplete, a good and suggestive outline of what lines of research may prove beneficial. Many of the tests are simple, but their interpretation is rather unsatisfactory. Others are so complicated that they could be carried out only with the facilities of a well equipped laboratory. Still others supply information which is as easily obtained by the ordinary bedside observation. There are a few tests, nevertheless, with whose technique the general practioner should be familiar, which are described in full. Among these may be mentioned the phenolphthalein test of kidney function and the adrenalin test for diabetes and Basedow's disease.

The book is interestingly written and suggestive of the work being done along those lines. The chapter on the ductless glands is particularly good. The impartial yet enthusiastic manner in which the various tests are presented make the book valuable as a book of reference on the subject. —A. A. G.

Compendium of Histo-Pathological Technic, by Emma H. Adler, Formerly Technician Pathological Laboratory, Presbyterian Hospital, New York. Cloth. 92 pages. Price, $1.25. Published by Paul B. Hoeber, New York, 1918.

Though there long has been a demand for this type of compendium, few publishers have had the courage to print them due to the cost and fear that their smallness might work against their popularity. The impression seems to prevail that only works voluminous in character are worth a physician's consideration. As a matter of fact, doctors will welcome just such a book as boils its subject down to the practical essentials and places at their disposal knowledge which, with little expense, may be used in their own office. As an aid in popularizing among general practitioners laboratory diagnostic methods, this book is a leader.
—A. A. G.

Personal Hygiene and Home Nursing, a Practical Text for Girls and Women for Home and School Use, by Louisa C. Lippitt, R.N., Assistant Professor of Correction Exercises, University of Wisconsin. (In New-World Science Series, edited by Professor John W. Ritchie.) Illustrated. Cloth. VII plus 256 pages. Price, $1.28. Published by World Book Company, Yonkers-on-Hudson, New York.

Simplicity and directness of style will insure this little voulme on popular nursing and hygiene an instant success. Though coming at a time when the market is full of books on the same subject, the attractive and practical manner of handling its material gives it a place apart from all others. Physicians, recommending it to mothers and girls entering into the age of womanhood, will find in it a valuable ally in preserving the health of their community. —A. A. G.

Manual on Physical Diagnosis, by W. D. Rose, M. D., Lecturer on Physical Diagnosis and Associate Professor of Medicine in Medical Department of The University of Arkansas. Published by C. V. Mosby Company, St. Louis, Mo. Price, $4.00.

This book will find a place among the many books written on the same subject. Its convenient size and durable binding are especially attractive. The many cuts scattered through the text prove very useful in further explaining the text. A. A. G.

